# 5-Aminolevulinic-acid-based fluorescence spectroscopy and conventional colposcopy for *in vivo* detection of cervical pre-malignancy

**DOI:** 10.1186/s12905-015-0191-4

**Published:** 2015-04-17

**Authors:** Rasa Vansevičiūtė, Jonas Venius, Olga Žukovskaja, Daiva Kanopienė, Simona Letautienė, Ričardas Rotomskis

**Affiliations:** National Cancer Institute, Vilnius, Lithuania; Laboratory of Biomedical Physics, National Cancer Institute, Vilnius, Lithuania; Faculty of Medicine, Vilnius University, Vilnius, Lithuania; Biophotonics Group, Laser Research Center, Vilnius University, Vilnius, Lithuania

**Keywords:** Cervical intraepithelial neoplasia; 5, Aminolevulinic acid, Fluorescence diagnostics, Fluorescence spectroscopy, Protoporphyrin IX

## Abstract

**Background:**

Sensitized fluorescence diagnostics are based on selective accumulation of photosensitizer in the tissue where carcinogenesis has started. The present study compared topical 5-aminolevulinic acid (5-ALA)-based fluorescence spectroscopy (FS) *in vivo* with conventional colposcopy for cervical intraepithelial neoplasia (CIN) detection.

**Methods:**

We enrolled 48 patients who were referred for colposcopy because of high-grade changes in cervical cytology. Every inspected cervix was divided in to quadrants, and there were 174 quadrants included in the study. Each patient had a cytological smear, colposcopy, FS and histopathological analysis. For FS, 3% 5-ALA cream was used topically and after an average 135 min incubation, fluorescence spectra were recorded from the cervix *in vivo*. FS and colposcopy results were correlated with histopathology.

**Results:**

All spectra were evaluated by a ratio of the protoporphyrin IX fluorescence intensity at 634 nm and autofluorescence intensity at 510 nm. For proper grouping of low-risk and high-risk cases, a threshold of 3.87 was calculated. Data per quadrant showed that FS had higher sensitivity than colposcopy (71.7% vs 67.4%) but specificity was greater for colposcopy (86.6% vs 75.6%). Combination of the methods showed higher sensitivity (88.0% vs 67.4%) but reduced specificity (88.0% and 69.5%), but it had the highest number of correctly identified high-risk changes and the highest (79.3%) accuracy. Data for each patient showed FS sensitivity of 91.2%, which was greater than for colposcopy (88.2%). Higher overdiagnosis resulted in decreased specificity for fluorescence methodology—71.4% versus 78.6% for colposcopy. In both cases, accuracy was 85.4% and effectiveness was >80%, which means that both methods can be used to determine high-risk cervical intraepithelial neoplasia. The diagnostic sensitivity of 97.1% for this complementary diagnosis indicates that it could be the best choice for detection of high-risk changes.

**Conclusions:**

5-ALA-based FS is an objective method, requiring short-term administration for appropriate fluorescence measurements. FS is a promising diagnostic tool with similar accuracy as colposcopy but with the potential advantage of providing objective results.

## Background

Worldwide, cervical cancer is the third most common cancer among women, with an age-standardized incidence rate of 15.3 per 100,000 and mortality rate of 7.8 per 100,000 [1]. Cervical cancer mostly affects younger women aged 35–50 [2]. In 2011, 452 new cases of cervical cancer were reported in Lithuania, which is one of the highest rates of morbidity for cervical cancer rate among the Baltic and Nordic countries [3,4].

Cervical intraepithelial neoplasia (CIN) is the potentially premalignant transformation and abnormal growth of epithelial cells on the surface of the cervix. A premalignant lesion is always at a risk of malignant transformation if stimulated by certain exogenous factors or conditions. The main factor directly related to CIN development is persistent human papillomavirus infection; mainly with high-risk types 16, 18, 31 and 33 [5,6]. If CIN is diagnosed at the appropriate time before cervical cancer manifestation, it may be cured and cervical cancer avoided [7,8].

The main disadvantages of contemporary CIN detection methods are high false-negative rates and low sensitivity of cytology and low specificity of colposcopy. Some studies showed that, in most cases, patients with high-grade smear results and high-grade impression on colposcopy have an acceptable overtreatment rate [9,10]. However conventional colposcopy demands long-term training and achieves an average ~48% (23–87%) specificity and has unsatisfactory accuracy even in trained hands [7,11-13]. The low positive predictive value seen with conventional colposcopy results in unwarranted surgical procedures and an additional burden on cervical cancer screening programs. These data suggest that there is a need for new diagnostic methods to improve or replace colposcopy for a clearer CIN diagnosis and more individual approach.

Recently, there has been increased interest in optical biopsy to determine pathological diagnoses in various organs. Optical biopsy refers to any technique that uses the interaction of light and tissue to provide information about tissue morphology without the need for excision [14-18]. Premalignant and malignant tissue differs from healthy tissue in its morphology and cell growth rate, which results in altered optical characteristics [16,19-21]. Most of the optical methods used in diagnostics are based on different types of spectroscopy: fluorescence, near infrared, Raman, diffuse reflectance, and similar techniques [22-25]. However, the most widely used techniques in clinical practice are based on fluorescence [26,27]. When light interacts with the molecules in the tissue they become excited and may re-emit light of different colors (fluorescence). The fluorescence of the tissue can be traced by sensitive spectrometers and provide characteristic spectra that reflect carcinogenicity of the tissue [22,23,27]. The acceptance and suitability of these methods clinically are determined by the diagnostic effectiveness, simplicity and low cost of the procedures. Moreover, they are noninvasive and can be repeated many times.

The potential of the fluorescence method to identify normal and pathological tissues of the uterine cervix was raised in 1994 [28,29]. The possibility was investigated of autofluorescence being used for detection of cervical neoplasia. Despite the demonstrated diagnostic possibilities, tissue spectra vary both among patients and within each individual patient. That complicates application of the method, and it is difficult to maintain the exact measurement conditions that could additionally influence the intensity and spectral variations [28]. The developed algorithms partially resolve this problem but the lack of sufficient contrast between fluorescence of healthy and neoplastic tissue encourages the use of sensitized fluorescence.

One of the most widely used precursor exogenous molecules in medical applications is 5-aminolevulinic acid (5-ALA). 5-ALA is a precursor of the fluorescent endogenous fluorophore, protoporphyrin IX (PpIX); an excess of which is produced in altered, especially cancerous tissue. This results in accumulation of intracellular porphyrins, which increases tissue fluorescence in the red spectral region of cancerous tissue [30]. Subsequent irradiation of the lesion with visible light matching the highest absorption of PpIX (~405 or ~630 nm) leads to red fluorescence emission from PpIX, peaking at 635 nm. The different accumulation of endogenous fluorophores in cancerous and normal tissue causes differences in the red fluorescence ratio between healthy and premalignant/malignant tissue, which makes detection and analysis by fluorescence light more applicable to discrimination between malignant and nonmalignant tissues [31]. This intensity is governed by the biological object itself, as well as many other conditions, such as variations in different preparations, doses and forms of application of 5-ALA, as well as different light sources and incubation times.

The aim of this research was to compare the ability of conventional colposcopy and 5-ALA-based FS for detection of CIN. From the fluorescence measurements, we established low-risk and high-risk tissues for cancer development. We created a methodology to identify from the fluorescence spectra a universal independent threshold value for determining high-risk tissues for cancer development, eliminating possible measurement variations, depending on individual characteristics of the organism and technical errors.

## Methods

The study was carried out at the National Cancer Institute in Lithuania between December 2012 and January 2014. The investigation protocol was approved by the Vilnius Regional Research Ethics Committee and written informed consent was obtained from all women. Inclusion criteria were nonpregnant women aged >18 years with suspected high-grade changes in cervical cytology, with no allergies or history of porphyria. Exclusion criteria were unsatisfactory or absent histopathological analysis, or inappropriate colposcopy or FS measurements.

**Figure 1:**
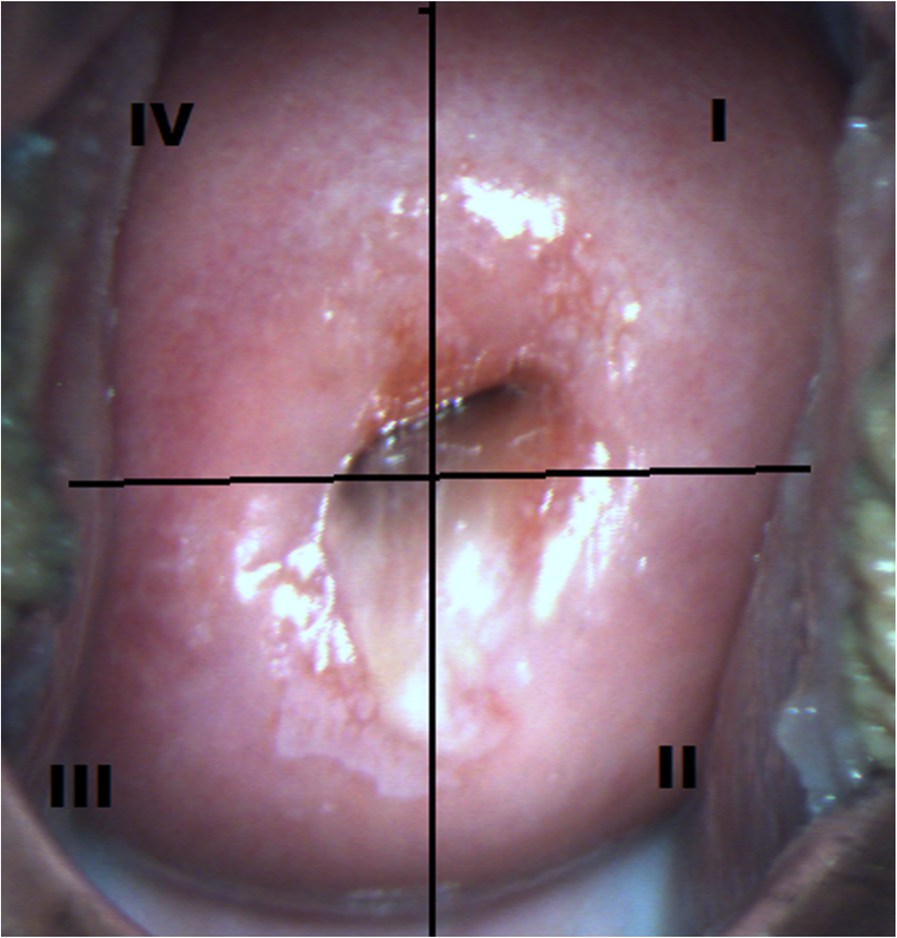
Colposcopic view of the cervix and conventional marking of the quadrants.

A total of 48 women with an average age of 36 years (range 23–57 years) were enrolled. Each patient underwent cytological smear, colposcopy, and FS. Every inspected cervix was divided into four quadrants clockwise (Figure 1), and every quadrant was analyzed as a separate case. In total, 48 patients and 174 cervical quadrants were included.

Data of 18 cervical quadrants were excluded owing to absence of histopathological analysis (uncertain marking or absence of the quadrants after loop excision or biopsy ([8] cases), coagulation defects ([5] cases)) and inappropriate FS measurements (bleeding from the cervical tissue during the investigation ([4] cases), device failure during measurement [1 case]).

After colposcopy and FS, punch biopsy or loop electrosurgical excision of the cervix for histopathological analysis was performed. These tissue samples were diagnosed as inflammatory (chronic cervicitis) (CIN0), mild (CIN1), moderate (CIN2) or severe (CIN3) neoplastic. All cases were divided in two clinically significant groups: low risk for cancer development (CIN0-1) and high risk for cancer development (CIN2-3). The histopathological diagnosis was provided for every quadrant of the cervix. However, the diagnosis of separate cervical quadrant (per quadrant) and subsequent final diagnosis of the whole cervix (per patient) was concluded according to the highest degree of the neoplasia discovered histopathologically.

**Figure 2:**
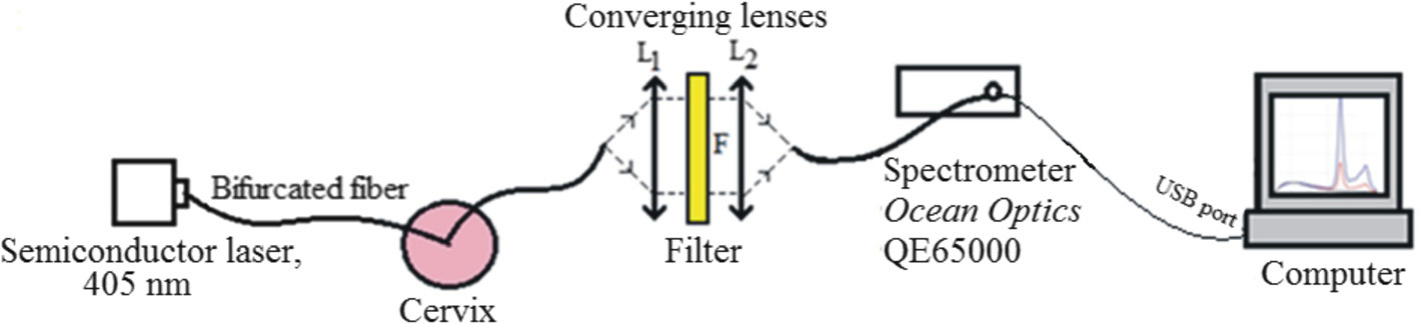
Scheme of the fluorescence spectroscopy system used for fluorescence photodetection of cervical lesions.

Conventional colposcopy was performed using 3% acetic acid with the Zoomscope Trulight video colposcope (Wallach Surgical). Lugol’s iodine was not used. The colposcopic examination was performed by two experienced specialists. FS measurements and analysis were performed with a spectroscopy system incorporating a 405-nm laser diode, optical fiber probe, and filter, and spectrometer QE65000 (Ocean Optics) was used to record fluorescence spectra of a cervix *in vivo* (Figure 2). Each woman received 3% 3 g 5-ALA cream (precursor of the endogenous fluorophore PpIX) topically as a visualizing agent on the cervix. The cream was prepared in a local pharmacy and used immediately afterwards. The incubation interval was chosen according to the published scientific data that have been shown to be diagnostically efficient [32-34]. The minimum incubation time was 90 minutes and the maximum was 180 minutes. The average and median of incubation time was 135 minutes.

**Figure 3:**
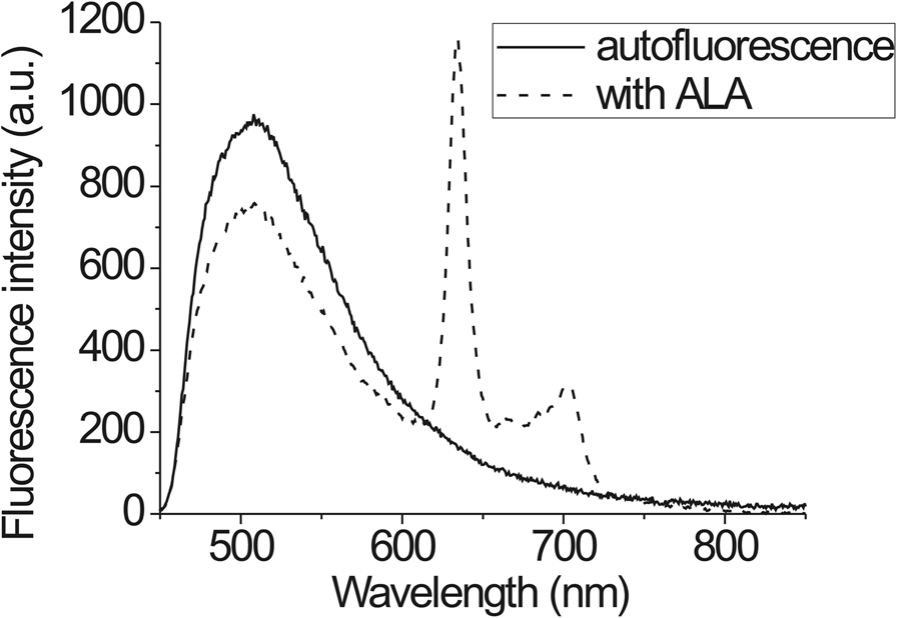
Fluorescence spectra from cervical tissue after ALA application with PpIX fluorescence maximum at 634 nm and autofluorescence maximum at 510 nm plus autofluorescence spectra of the cervical tissue.

The fluorescence spectra measurements of cervical tissues were done before 5-ALA application and after a certain incubation time (Figure 3). The study was carried out in a darkened room. During the examination, fluorescence spectra were acquired from 3–5 sites of every cervical quadrant. Additional spectra corresponding to healthy cervical tissue were registered from places that were not typical of neoplastic development and that had no signs of inflammation. All fluorescence spectra were processed using Origin Pro software.

The fluorescence intensity ratio (R) was evaluated from fluorescence spectra of sensitized cervical tissue (1):

1 $$ R=\frac{I\left(634 nm\right)}{I\left(510 nm\right)} $$

where I_(634 nm)_ is fluorescence intensity at 634 nm (maximum of the fluorescence band of the endogenous fluorophore PpIX) and I_(510 nm)_ is at the tissue autofluorescence intensity maximum at 510 nm. Our previous studies revealed that it is not correct to determine the true state of the quadrant by averaging all fluorescence spectra measured from that quadrant [35]. Therefore, diagnosis according to fluorescence data was made on the basis of one fluorescence spectrum that had the highest R value. This value should correspond to the highest degree of neoplasia.

In our previous study, we observed that R values are scattered among patients [35], and the reliable threshold value for healthy and pathological tissue differentiation could not be estimated. Therefore, considering that every patient has individual fluorophore composition and metabolic characteristics (depending on age, menstrual cycle, metabolic and endocrine functions) that influence the intensity of fluorescence spectra [20,36-38], the spectral measurements were additionally normalized for every patient. The normalized value D, diagnostic factor, was calculated as the proportion of R from individual quadrant and R from – healthy tissue of the particular women cervix (2):

2 $$ D=\frac{R(quadrant)}{R(healthy)} $$

where D is diagnostic factor, R_(quadrant)_ is individual quadrant, and R_(healthy)_ is healthy tissue of the particular cervix.

## Statistical analysis

Using Origin Pro 8, we performed statistical analysis of the D values for all quadrants. To determine the normality of the distribution, we applied the Shapiro–Wilk test, when the significance level was 0.05. We identified that our data were not distributed normally, so for further analysis, we applied nonparametric tests. To inspect the raised hypotheses, we performed the Mann Whitney Wilcoxon *two-independent-samples t*ests. The statistically significant difference was when *P*(H_0_ = 0) < 0.05. For proper grouping of LR and HR cases (D values), we calculated the threshold value. For this reason Youden’s index (J) was assessed. According to this criterion, the threshold value is when J is the greatest. Statistical analysis of the collected material included sensitivity, specificity, positive and negative predictive values for FS, colposcopy and combination of those methods, analyzing diagnosis per patient and per cervical quadrant. For evaluation of the method diagnostic efficiency, receiver operating characteristic curves (ROCs) were plotted. The diagnostic value was obtained by assessing area under the curve (S_ROC_). Area under the curve was 0.9–1.0, the study could be evaluated as excellent; 0.8–0.9, very good; 0.7–0.8, good; 0.6–0.7, tolerable; and 0.5–0.6, deficient [39,40]. ROC analysis was performed using MedCalc.

## Results

### FS data analysis

**Figure 4:**
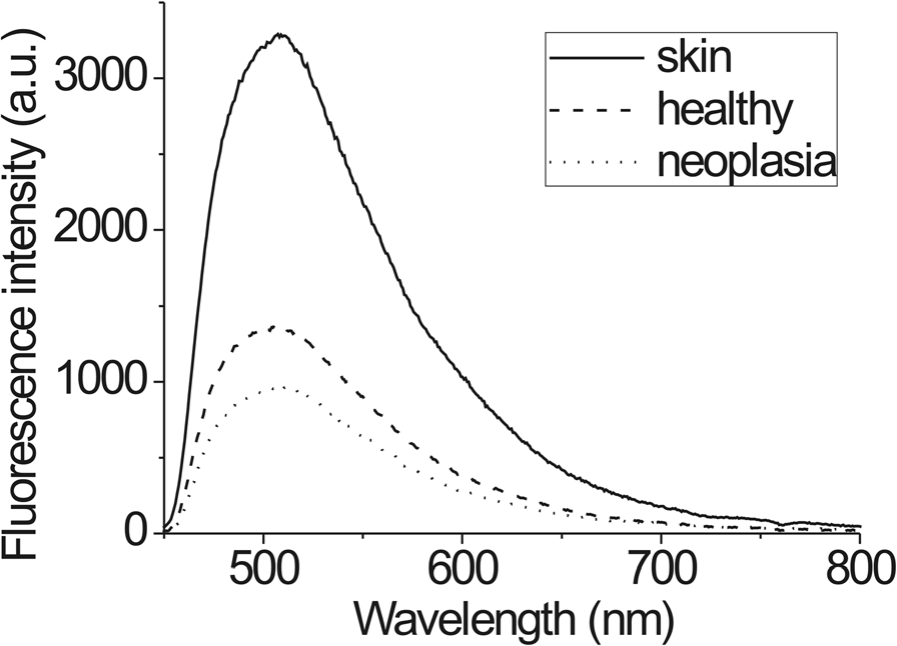
Fluorescence spectra from neoplastic, healthy cervical tissue and skin before ALA application in the same patient.

**Figure 5:**
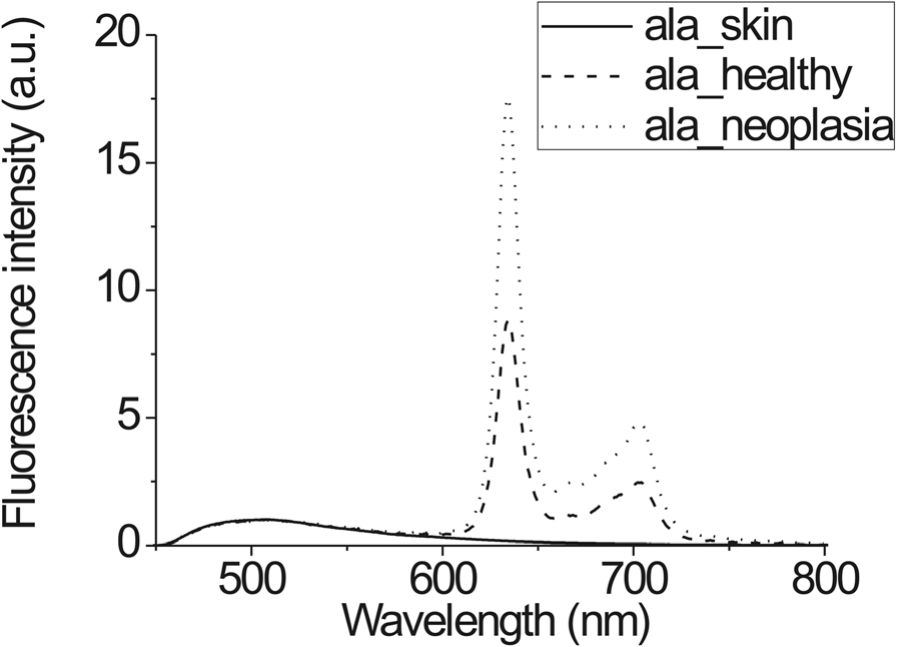
Fluorescence spectra from neoplastic, healthy cervical tissue and skin after topical ALA application in the same patient.

During examination, 1295 *in vivo* spectra were acquired (583 before 5-ALA application and 712 after). Before 5-ALA application, no notable PpIX fluorescence was observed in the cervical tissue. Typical fluorescence spectra of different tissue types are presented in Figure 4. After topical application of 5-ALA, PpIX fluorescence was detected in the spectra of healthy and pathological tissues (peaks at 634 and 703 nm). The typical fluorescence spectra of the cervical tissues, normalized to the maximum of the tissue autofluorescence are presented in Figure 5.

After histopathological analysis, we diagnosed 28% and 47% of low-risk cases and then 71% and 53% of high-risk cases per patient and per cervical quadrant, respectively (Table 1).

**Table 1:** **Histopathological diagnosis of analyzed cases**

**Diagnosis (histopathology)**		**Total per patient**	**Total per cervical quadrant**
		**n = 48**	**n = 174**
CIN 0	LOW RISK	11 (23%)	80 (46%)
CIN 1		3 (6%)	2 (1%)
CIN 2	HIGH RISK	2 (4%)	7 (4%)
CIN 3		32 (67%)	85 (49%)

The higher fluorescence intensity in the red spectrum region was detected from neoplastic tissue compared with healthy tissue. Normalization of fluorescence spectra to autofluorescence peaks helps to overcome varying measurement conditions and allow unambiguous evaluation of fluorescence spectra from different places. The spectrum measured on the skin did not have a peak specific for PpIX, which means that ALA cream used topically on the cervix causes no PpIX production in other parts of the body, showing no systemic accumulation and therefore causing no notable side effects.

### Calculation of the threshold value

**Figure 6:**
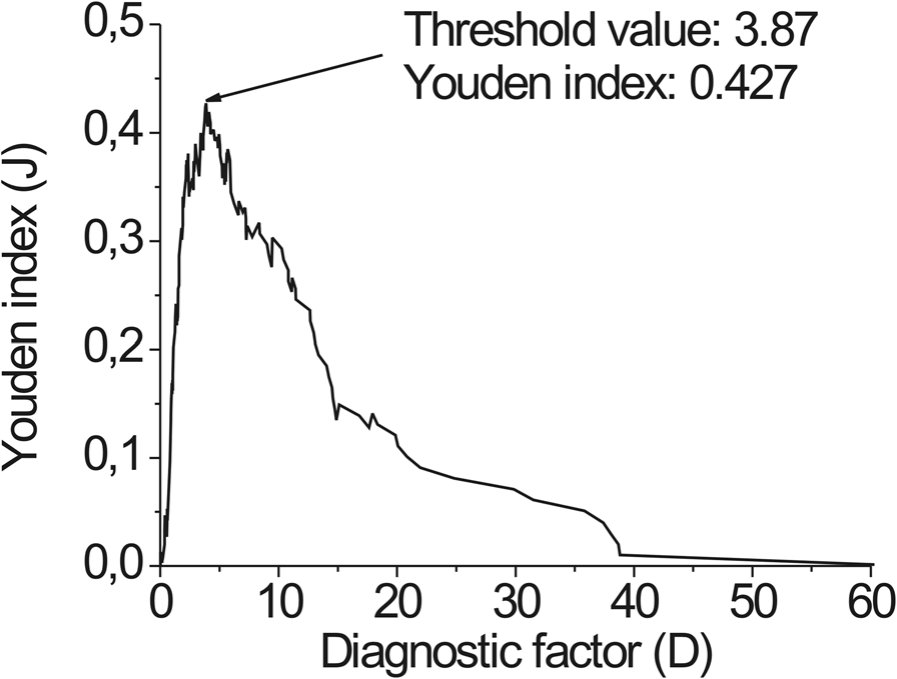
Youden’s index in dependence of optimal threshold value.

To obtain the threshold value for low- and high-grade differentiation, D value was calculated for every quadrant and Youden’s index was calculated for every D value (Figure 6). The peak value of Youden’s index was 0.427, which was reached at a D value of 3.87. This value was chosen as the threshold value for low-risk and high-risk cervical tissue differentiation. Subsequently, D values split into two groups (n_(CIN0-1)_ = 88; n_(CIN2-3)_ = 86). After statistical analysis using the Shapiro–Wilk test, at the 0.05 level of significance, the D values of both groups were not normally distributed. To establish whether these two groups were statistically different, the Mann Whitney Wilcoxon test was performed for independent samples and showed at the 0.05 level of significance that the D values of the HR group were higher than those of the LR group, *P(H*_0_ = *0)* = 2,53 × 10 ^9^. After FS and D factor calculation, cervical quadrants, where D was >3.87, were diagnosed as having high-risk neoplasia, and all other quadrants were classified as low risk.

### Feasibility of FS for high/low risk cervical neoplasia diagnosis

Further analysis was performed to evaluate the possibility of using FS for differentiation of high- and low-risk lesions. FS and colposcopy data were compared with the gold standard of histopathological diagnosis (Table 2). It was obvious that, based on fluorescence data, 20 low-risk quadrants were mistakenly diagnosed as high-risk CIN, and in 26 quadrants, no pathological changes were seen, and high-risk CIN was demonstrated histopathologically.

**Table 2:** **Comparison of fluorescence spectroscopy and colposcopy with histopathology (per quadrant)**

**Histopathology**				
		**HR**	**LR**	**Total**
Fluorescence spectroscopy	HR	66	20	86
	LR	26	62	88
	Total	92	82	174
Fluorescence spectroscopy + colposcopy	HR	81	25	106
	LR	11	57	68
	Total	92	82	174
Colposcopy	HR	62	11	73
	LR	30	71	101
	Total	92	82	174

In determining the status of the cervical quadrants by colposcopy, 73 quadrants were high-risk and 101 were low-risk neoplastic. According to these results, the disease was missed in 30 quadrants, while only 11 cases were incorrectly diagnosed as high risk.

One of the potential applications of fluorescence diagnostics is as a complementary method to conventional techniques. High-risk neoplasia was considered if at least one method detected neoplastic changes. The data in Table 2 show that when colposcopy and fluorescence were combined, only 11 cases were missed, and combination of methods gave the best results. However, the 25 low-risk quadrants were mistakenly identified as high risk, which led to unnecessary surgical procedures.

Following these results, the sensitivity, specificity, accuracy, test value, and positive and negative predictive values for FS, colposcopy and combination of the methods were calculated. Table 3 represents the statistical data for the cervical quadrants (per quadrant). FS had higher sensitivity than colposcopy (71.7% vs 67.4%), but specificity was higher for colposcopy (86.6% vs 75.6%). The combination of methods had higher sensitivity (88.0% vs 67.4%) but reduced specificity (88.0% vs 69.5%). As mentioned above, combination of the methods resulted in the highest number of correctly identified high-risk changes and the highest accuracy (79.3%). According to the ROC curves, the diagnostic values (S_ROC_) were similar and could be considered as good. The effectiveness of the combined diagnostic methods was the best (S_ROC_ = 0.788).

**Table 3:** **Sensitivity, specificity, accuracy, positive and negative predictive values for fluorescence spectroscopy, colposcopy and combination of methods, analyzing cervical quadrant diagnosis**

	**Fluorescence spectroscopy**			**Colposcopy**			**Colposcopy + fluorescence (combined)**		
	**n**	**%**	**95% CI**	**n**	**%**	**95% CI**	**n**	**%**	**95% CI**
Sensitivity	66/92	71.7	61.4- 80.6	62/92	67.4	56.8- 76.8	81/92	88.0	79.6- 93.9
Specificity	62/82	75.6	64.9- 84.4	71/82	86.6	77.3- 93.1	57/82	69.5	58.4- 79.2
Accuracy	128/ 174	73.5	-	133/174	76.4	-	138/174	79.3	-
PPV	66/86	76.7	-	62/73	84.9	-	81/106	76.4	-
NPV	62/88	70.5	-	71/101	70.3	-	57/68	83.8	-
Total	174			174			174		

After quadrant analysis, data were summarized to provide the general diagnosis for the patient. The quadrant with the severest neoplasia determined the diagnosis of the whole cervix. Comparison of fluorescence and colposcopy data for each patient with the histopathological findings are presented in Table 4.

**Table 4:** **Comparison of fluorescence spectroscopy and colposcopy with histopathology (per patient)**

**Histopathology**				
		**HR**	**LR**	**Total**
Fluorescence spectroscopy	HR	31	4	35
	LR	3	10	13
	Total	34	14	48
Fluorescence spectroscopy + colposcopy	HR	33	6	39
	LR	1	8	9
	Total	34	14	48
Colposcopy	HR	30	3	33
	LR	4	11	15
	Total	34	14	48

Per patient analysis showed that FS failed to detect only three high-risk cases from 34, while during colposcopy, four cases were missed. However, colposcopy overdiagnosed three patients and FS four. These results show that the two methods are comparable. Combination of the methods increases the number of correctly diagnosed neoplasia (Table 4) and only one high-risk case was missed. However, overdiagnosis also increased, with six patients being mistakenly diagnosed with high-risk changes.

### Full statistical analysis for the per patient results is presented in Table 5

**Table 5:** **Sensitivity, specificity, accuracy, positive and negative predictive values for fluorescence spectroscopy, colposcopy and combination of methods, analyzing per patient diagnosis**

	**Fluorescence spectroscopy**			**Colposcopy**			**Colposcopy + fluorescence (combined)**		
	**n**	**%**	**95% CI**	**n**	**%**	**95% CI**	**n**	**%**	**95% CI**
Sensitivity	31/34	91.2	76.3- 98.0	30/34	88.2	72.5-96.6	33/ 34	97.1	84.6- 99.5
Specificity	10/14	71.4	41.9- 91.4	11/14	78.6	49.2-95.1	8/14	57.1	28.9- 82.2
Accuracy	41/48	85.4	-	41/48	85.4	-	41/ 48	85.4	-
PPV	31/35	88.6	-	30/33	90.9	-	33/ 39	84.6	-
NPV	10/13	76.9	-	11/15	73.3	-	8/9	88.9	-
Total	48			48			48		

Sensitivity of FS was 91.2%, which was greater than 88.2% for colposcopy. Greater overdiagnosis resulted in decreased sensitivity for FS—71.4% vs 78.6% for colposcopy. In both cases, the accuracy was 85.4%, and the effectiveness (S_ROC_) was >80%, which means that the value in determining the high-risk CIN was good for both methods. On the contrary, the effectiveness of the combined methods was <80%. Nevertheless, the sensitivity of this combined diagnosis was 97.1%, indicating that it was best for detection of high-risk lesions.

## Discussion

We evaluated the use of 5-ALA-based FS for the detection of pathological areas in the cervix. Identification of the diseased areas was estimated by measuring the fluorescence of PpIX, and more quantitative evaluation could be done by calculating the ratio (R) between porphyrin fluorescence and tissue autofluorescence. The additional normalization of ratios (R) for the inspected areas of the tissue to the (R_healthy_) value of healthy tissue enabled us to calculate the diagnostic coefficients (D), and to obtain threshold values for low- and high-risk differentiation. The threshold value of 3.87 is the universal independent number that could be used for CIN diagnosis.

Examination of the cervical quadrants yields the possibility of specifying the localization of neoplastic regions. However, because they were observed during the study, the pathological areas could be small and missed during measurement. Therefore, for precise localization of all lesions, careful and fine scanning should be performed. In other cases, accurate diagnostics could only be performed per patient. The results of FS per quadrant in some cases were influenced by difficulties during conization, which resulted in incorrect margins of the quadrants and mixed correct results. However, during routine procedures, such situations cannot be fully avoided, therefore, such inaccuracies must be incorporated when calculating the sensitivity and specificity as a random error. Nevertheless, these effects do not influence per patient diagnosis.

Per patient diagnosis showed 85.4% accuracy of FS in identifying neoplastic changes, therefore, it has great diagnostic potential. However, to replace the conventional methods, the threshold value must be revised and, if necessary, corrected after data analysis from more patient cases.

Several studies show that the detection of porphyrin fluorescence can improve the identification of the cervical pre-malignancy. Only a few studies have been performed using FS with 5-ALA. Hillemanns *et al*. [32] showed that fluorescence imaging with 5-ALA after 60–90 minutes achieved similar sensitivity and specificity compared with colposcopy in detecting CIN: 94% and 51% versus 95% and 50%, respectively. However, the specificity was markedly improved by FS, achieving 75%. Sapoznikova *et al.* [41] reported that sensitized FS had a diagnostic efficiency of 79.5% for cervical neoplasia.

When comparing colposcopy with 5-ALA-based FS, on the one hand we have the experience of physicians and on the other hand, we have the objectivity of the diagnostic method, and one also should decide between overestimated and underestimated CIN diagnosis. To date, it seems that this should be a compromise because there is still no single method or combination of methods that can perfectly fulfill all the desired requirements.

Nevertheless, fluorescence diagnostics appears promising despite non- ideal results. Fluorescence is an objective method, requiring only a short training for appropriate fluorescence measurements, while colposcopy is highly dependent on physicians’ experience and might be more inaccurate when performed by less-trained hands. There is plenty of room for improving the precision and consistency of fluorescence measurement techniques. The additional burden for FS was because the analysis was performed on patients who already had high-grade cytology results. Altered cytology could determine greater production of PpIX, which results in elevated fluorescence intensity and finally provide false-positive results and decreased specificity. Moreover, the women who were diagnosed as having no evidence or low risk of neoplasia had significant signs of inflammation. The inflammation is not classified as malignancy, but it changes the optical properties of cervical tissue, therefore, the ability to differentiate inflammation from neoplastic changes should further increase the diagnostic value of FS.

A possible clinical application could be a combination of a porphyrin fluorescence image viewer and an *in vivo* spectrum analysis system, which could be used for optical biopsies. With this system, high-grade pre-cancer could be diagnosed and treated in one session [32-34,41].

It is possible to define further directions for development. FS is a powerful technique for detecting altered tissues, however, additional attention should be paid to differentiation/identification of the neoplastic changes and inflammation.

## Conclusions

By standardization of instrumentation and proper diagnostic algorithms it was possible to obtain an independent threshold value for low- and high-risk differentiation, D_threshold_ = 3.87. Analysis using this threshold showed the potential of FS for noninvasive identification of neoplastic changes at an early stage, and it could be used for differentiating high-risk changes. This method shows similar accuracy to currently used screening tests, but has the potential advantage of objective results. FS requires only a short time of training to achieve appropriate measurements, while colposcopy is highly dependent on physicians’ experience, which is usually considered a major factor in its success.
